# Mistreatment during childbirth and postnatal period reported by women in Nepal —a multicentric prevalence study

**DOI:** 10.1186/s12884-022-04639-6

**Published:** 2022-04-14

**Authors:** Rejina Gurung, Md Moinuddin, Avinash K. Sunny, Amit Bhandari, Anna Axelin, Ashish KC

**Affiliations:** 1 Research Division, Golden Community, Lalitpur, Nepal; 2 Department of Women’s and Children’s Health, Uppsala University, Uppsala, Sweden; 3 Edge Hill University Medical School, Ormskirk, UK; 4 Institute of Child Health, University College London, London, UK; 5 Society of Public Health Physician Nepal, Kathmandu, Nepal; 6 Department of Nursing Science, University of Turku, Turku, Finland

**Keywords:** Mistreatment during childbirth, Respectful care at birth, Disadvantaged ethnic group, Health system and Nepal

## Abstract

**Introduction:**

Trust of women and families toward health institutions has led to increased use of their services for childbirth. Whilst unpleasant experience of care during childbirth will halt this achievement and have adverse consequences. We examined the experience of women regarding the care received during childbirth in health institutions in Nepal.

**Method:**

A prospective cohort study conducted in 11 hospitals in Nepal for a period of 18 months. Using a semi-structured questionnaire based on the typology of mistreatment during childbirth, information on childbirth experience was gathered from women (*n* = 62,926) at the time of discharge. Using those variables, principal component analysis was conducted to create a single mistreatment index. Bivariate and multivariate linear regression analyses were conducted to assess the association of the mistreatment index with sociodemographic, obstetric and newborn characteristics.

**Result:**

A total of 62,926 women were consented and enrolled in the study. Of those women, 84.3% had no opportunity to discuss any concerns, 80.4% were not adequately informed before providing care, and 1.5% of them were refused for care due to inability to pay. According to multivariate regression analysis, women 35 years or older (β, − 0.3587; *p*-value, 0.000) or 30–34 years old (β,− 0.38013; *p*-value, 0.000) were less likely to be mistreated compared to women aged 18 years or younger. Women from a relatively disadvantaged (Dalit) ethnic group were more likely to be mistreated (β, 0.29596; *p*-value, 0.000) compared to a relatively advantaged (Chettri) ethnic group. Newborns who were born preterm (β, − 0.05988; *p*-value, 0.000) were less likely to be mistreated than those born at term.

**Conclusion:**

The study reports high rate of some categories of mistreatment of women during childbirth. Women from disadvantaged ethnic group, young women, and term newborns are at higher risk of mistreatment. Strengthening health system and improving health workers’ readiness and response will be key in experience respectful care during childbirth.

**Supplementary Information:**

The online version contains supplementary material available at 10.1186/s12884-022-04639-6.

## Background

Since the start of the millennium, globally, number of institutional births increased from 30 million to 80 million due to better care than home birth [1]. The proportion of women delivering in health institutions has increased by 7.8% in 2000 [2]. Poor care during and around childbirth attributed to 1 million stillbirths and 1.2 million neonatal deaths in 2019 [3]. Despite increased accessibility to institutional births in the last two decades, there has been evidence of discrimination in providing care based on the race, ethnicity and socio-demographic characteristics of the women. There has been high rate of unconsented care provided to younger and uneducated women during childbirth [4–6]. In the last decade, there has been widespread reporting of mistreatment of women during childbirth and postnatal period at health institutions [4]. And the mistreatment ranges from physical abuse and nonconfidential care to abandonment and detention in health facilities due to lack of payment [5, 6]. This disrespectful and abusive care will reduce the trust of women and families in health service utilization [7, 8].

A study in health institutions in Iran showed that even though the Health Care Professionals (HCP) acknowledged disrespectful care as a violation of human rights, they perceived certain disrespectful practices were intended to ensure the safety of mother and baby [9]. This reflects underlying gender-related notion and knowledge gap of operational definition of Respectful Maternity Care (RMC) among HCP. Recently, there has been recognition of adverse effects of disrespect and abuse on women, babies, including care providers’ job satisfaction [9].

A meta-analysis of seven studies conducted in Ethiopia showed that almost half of women experienced mistreatment during childbirth and maternity care [10]. The most common forms of mistreatment are physical abuse, nonconfidential care, and abandonment during childbirth [10]. A meta-analysis of 11 studies done in India showed that mistreatment during childbirth ranged from 10 to 77.3%, with lack of respect and dignity being the most frequent form of mistreatment [11]. The poor experience of care might be due to poor training and supervision of HCPs, as well as a lack of accountability of health institutions. HCPs who express their disrespect of women during childbirth are themselves disempowered within the hierarchy of the health system, are often low paid, and might experience mistreatment at home as women [12]. Overall, mistreatment during childbirth can result in the low utilization of health institutions by women and families.

The World Health Organization (WHO) envisions a world free of abuse and disrespect to women and babies during childbirth [13]. The WHO issues guidelines and standards for improving the quality of women’s and babies’ care around the time of birth [14]. Given the widespread reporting on the violation of women’s right to respectful care, the White Ribbon Alliance established a renewed focus on respect in maternity care with a 2019 charter [15].

In Nepal, Every Newborn Action Plan lays a plan to improve the provision and women’s experience of intrapartum care such that preventable maternal and neonatal mortality and stillbirth are prevented by 2030 [16]. During the last two decades, there has been an unprecedented increase in health institution childbirth in Nepal. Almost two thirds of women gave birth at different levels of health institutions in 2019 [17]. Provision of care concerning inadequate supply of equipment and drugs during childbirth has been overtly evident in the health facilities [18]. However, there is a paucity of evidence on the magnitude of mistreatment of women during childbirth in Nepal.

In this study, we aimed to measure the magnitude of mistreatment of women during childbirth in health institutions in Nepal.

## Methods

The study has been reported as per the checklist for Strengthening the Reporting of Observational Studies in Epidemiology (STROBE) [19].

### Design

We conducted a prospective cohort study in 11 public hospitals in Nepal for a period of 18 months (April 2017–October 2018). The study was nested within a larger study of 11 hospitals in Nepal that was evaluating the impact of a quality improvement package for neonatal resuscitation care on perinatal outcomes [20, 21].

### Setting

The study was conducted in 11 public hospitals of Nepal which are distributed in six out of seven provinces of Nepal, providing tertiary level of basic, emergency and comprehensive maternity care. Hospitals were selected based on the criteria with deliveries more than 1000 per year and referral centers for maternal and newborn care. All the hospitals provided normal vaginal, assisted vaginal and cesarean section delivery series hospital 1 in province 1, hospital 2 and 3 in Bagmati province, hospital 4, 5, 6, 7, 8 and 9 in Lumbini province, hospital 10 in Karnali province and hospital 11 in Sudurpachim province. All the hospitals, despite mostly being in the flat lands, were different in terms of service coverage and diverse in relation to ethnicity, language and religion. The hospitals vary in terms of service delivery as well as in serving ethnic minorities, population age groups, social norms and cultural practices. All the study hospitals have waiting room for women to receive care during labour until they are shifted to delivery room for normal, assisted or maneuver vaginal deliveries where immediate neonatal resuscitation service is available at birth. The labour unit and the events are managed by with team of obstetricians, pediatricians, medical officer and nurses. Most of the nurses working in the labour room are trained as Skilled Birth Attendants (SBAs) to manage normal and complicated deliveries under the supervision of obstetricians. They are also trained on providing essential newborn care services.

### Study participants

All women who were admitted to the 11 hospitals for childbirth during the study period were eligible to be included in the study. Women who consented to the study were enrolled, along with their newborns.

### Sample size

This was a nested study of a large observational study to evaluate the impact of a quality improvement intervention on perinatal care [22]. For the larger study, an estimated 80,000 women–baby pairs were required to assess the change in intrapartum-related mortality.

### Data sources

An independent and trained team of research nurses under the supervision of a research site coordinator was recruited in each hospital. To address varied ethnicity, language and culture among different geographical distributions of hospitals, surveillance officers were selected from the local applicants. Medical, obstetric and neonatal information of all the women enrolled in the study were extracted from the medical record journal while information on socio-demographic and childbirth experience were gathered during the interview conducted before discharge from the hospital. Postnatal women were interviewed using a semi structured questionnaire in a private and comfortable space using their preferred language inside the postnatal ward [20]. Interview was conducted after doctor’s round and in the absence of health care providers.

### Data management

After the completion of interviews, research site coordinator reviewed the forms, on a daily basis and maintained a surveillance form. Before indexing these forms in a sealed envelope, any discrepancies noticed by the coordinator were discussed with the data collector. The data-entry assistant then reviewed the forms received from each hospital for completeness, coded the open-ended questions (caste, location), and forwarded the forms to data-entry operators who entered the data in Census and Survey Processing System (CS PRO) database. The database manager reviewed the entered data of each hospital and backed up the data on a weekly basis in an external backup system in SPSS format.

### Variable definition

- Mistreatment of women and newborns is the deviation in the standards of care including any events of childbirth experienced as or intended to be disrespectful. We reviewed literature of typology proposed by Bowser and Hill [6] and WHO’s 2016 “Standards for improving quality of maternal and newborn care in health facilities” to develop the questionnaire [14]. A set of 12 questions to assess mistreatment of women and newborns during childbirth and postnatal period were developed based on the mistreatment typology proposed by Bohren and colleagues [5] (Table 1). Consultative workshop was held with experts in the quality of care to develop the questionnaire and a formative assessment for content validity was done.
- Women’s ethnicity was categorized into Brahmin/Chettri as relatively advantageous, janjati, madeshi, muslim, dalit and other as disadvantageous group.
- Women’s literacy was categorized as those who cannot read and write and those who can read and write,
- Women’s age was categorized as 18 years or less, 19–24 years, 25–29 years, 30–34 years and 35 years or more,
- Parity: Women who had no previous viable birth (0 parity), one previous birth (1 parity) and 2 or more previous birth (2 or more parity),
- Preterm birth: Birth of the baby with gestational age less than 37 weeks. The variable is categorized as less than 37 week and 37 weeks or more,
- Low birth weight: Infants with birth weight less than 2500 g.

**Table 1:** Mistreatment typology, index, and question variable

Mistreatment typology	Index	Questions/indicators
Abuse (physical, verbal, and sexual)	Abuse during labor or birth	Question 285. Were you or your newborn physically, verbally, or sexually abused during labor or childbirth or after birth?
Stigma and discrimination	Religious/cultural standards not met	Question 284. Did the health service meet your religious and cultural birthing practice needs?
Failure to meet professional standards of care	Not staying at least 24 h	Question 241. Did you stay at the health facility for at least 24 h after an uncomplicated vaginal birth?
	Baby not examined in presence of women	Question 276. Did a medical doctor examine your baby while you were present?
	Baby not examined before discharge	Question 277. Did a health worker examine your baby before discharge?
	Not adequately informed on the care provided	Question 280. Were you adequately informed by the care provider about examinations, actions, and decisions taken for your care?
	Refusal of care due to inability to pay	Question 287 Were you refused care because of inability to pay?
Poor rapport between women and providers	Not counseled on maternal danger signs	Question 272. Did the health worker counsel you on danger signs of the mother during delivery and postnatal period?
	Not counseled on neonatal danger signs	Question 274. Did the health worker counsel you on danger signs of baby during delivery and the postnatal period?
	Not counseled before discharge	Question 278. Before discharge, did you receive counseling from a skilled health service provider?
	Not counseled on exclusive breastfeeding	Question 248. Did you receive written or verbal information and counseling on exclusive breastfeeding until 6 complete months before discharge?
	No opportunity to discuss any concerns	Question 279. Were you given the opportunity to discuss any concerns and preferences?

### Data processing and statistical analysis

#### Data exploration and preprocessing

In total, there were 12 questions to measure the mistreatment of mothers and newborns during childbirth and the postpartum period. Options proposed for the response of all the questions were similar as “Yes”, “No”, “Don’t know” and “Not Available”. The proportion of each response was analyzed and presented in Table 2. We found out that the proportion of “Yes” response in some items was 99%; while the response rate was less than 10% in some items. Defining mistreatment based on the “Yes” response of any of these 12 questions might overestimate the condition, likewise, key information might be lost if the responses are combined into a binary variable. To overcome this issue, we constructed a continuous score to represent the mistreatment index using Principal Component Analysis (PCA) of the 10 variables [23]. Two variables were excluded due to the response rate less than 10% having very low variance. PCA is a dimension reduction technique used for combining many variables into a single continuous score [24]. A continuous score is more flexible to analyze and model (Fig. 2).

**Table 2:** Magnitude of mistreatment during childbirth and postnatal period reported by women

Mistreatment variables	No	Yes	Not applicable	Don’t know
Abuse during labor or birth	99.2	0.8	0	0.0
Religious/cultural standards not met	9.1	90.9	0	0.0
Discharge less than 24 h after childbirth	49.1	50.9	0	0.0
Baby not examined in the presence of the women	58.8	40.6	0	0.7
Baby not examined before discharge	39.5	59.9	0	0.6
Not adequately informed on care provided	80.4	19.6	0	0.0
Not counseled on maternal danger signs	28.6	66.3	0	5.1
Not counseled on neonatal danger signs	24.1	73.7	0	2.3
Not counseled before discharge	24.6	75.4	0	0.0
Not counseled on exclusive breastfeeding	57.9	42.1	0	0.0
No opportunity to discuss any concern	84.3	15.7	0	0.0
Refusal to care due to inability to pay	92.4	1.5	0	6.1

**Fig. 1:**
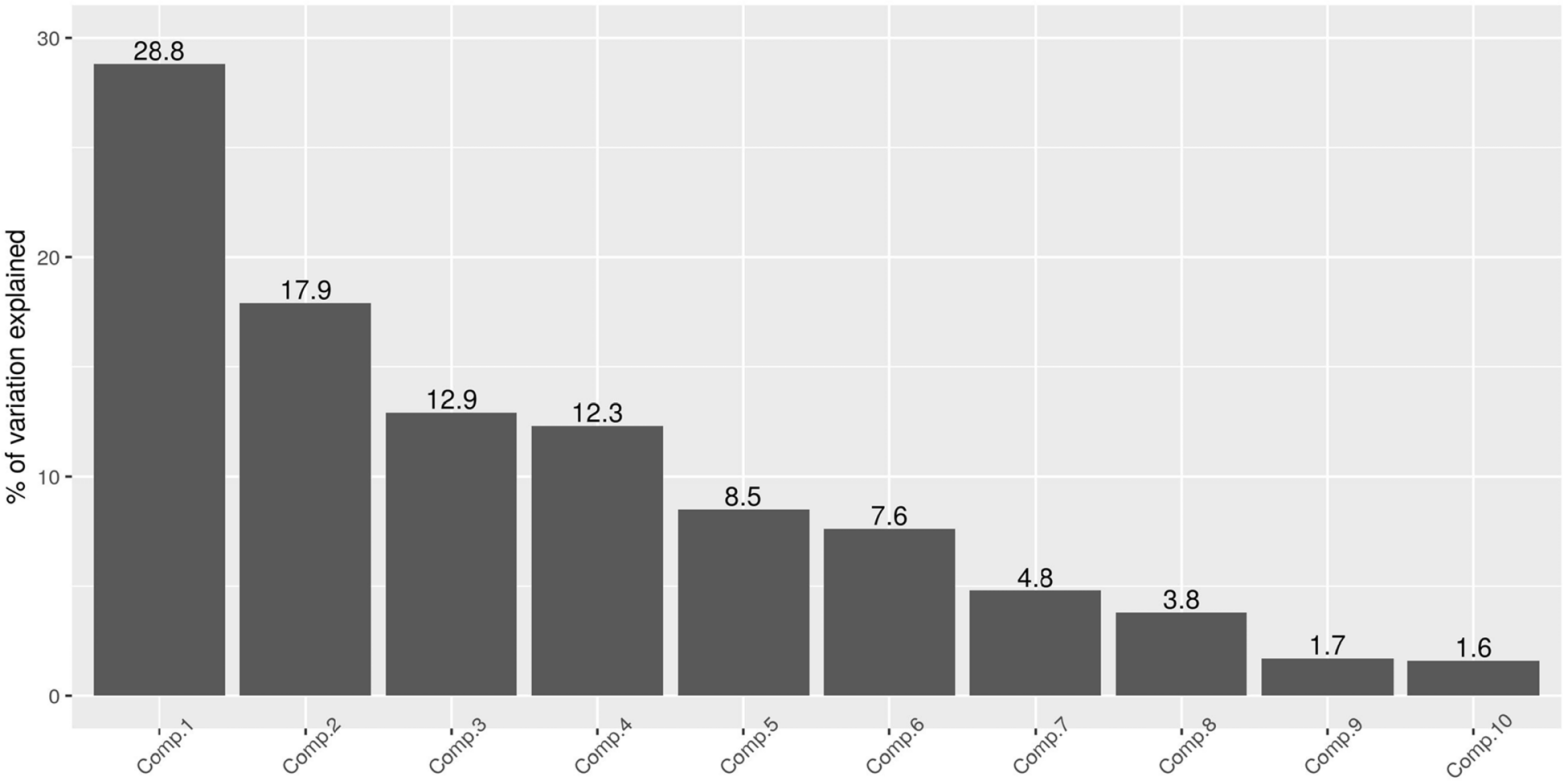
The percentage of variation explained by each principal component

We considered the first principal component as the proxy for the mistreatment index, which is our target variable. From now on, first principal component is labelled as PCA1. Note that the first component alone explains 29% of the total variation in the original 10 items (Fig. 1).

From Table 2, we see that there is a reasonable proportion of responses in the “Don’t know” category, which accumulates to 17.3%. This posed another challenge in creating the combined proxy index of mistreatment. These respondents fall into neither the “Yes” nor the “No” categories. We checked their sociodemographic characteristics and found that the respondents in the “Don’t know” category were approximately similar to those in the “Yes” or “No” category. Therefore, we excluded these observations when conducting regression analysis because the number of observations was sufficient to continue with further analysis.

Another possibility is to impute the missing values. However, we did not adopt that strategy because there is a possibility of a spurious association between the imputed values and the characteristics used to impute the values.

#### Statistical analysis

The data were described using suitable summary statistics such as proportions for the categorical responses. For readability, the data summary is presented in graphs. The raw associations between the outcome and potential correlates were assessed using graphical tools and simple linear regression models of PCA1 on each of the covariates. The potential sociodemographic characteristics associated with mistreatment we considered, based on the previous literature, were age, education, ethnicity, and parity of the mothers as well as sex and preterm status of the baby. The significant covariates at the 10% level were considered for inclusion in the multiple regression model. The multiple regression model was fitted determine out the correlates of mistreatment among women during delivery after adjusting for the potential confounding effects by other covariates.

All of the data analysis was conducted using R version 3.6.2 on the Linux operating system.

## Results

### Participants

**Fig. 2:**
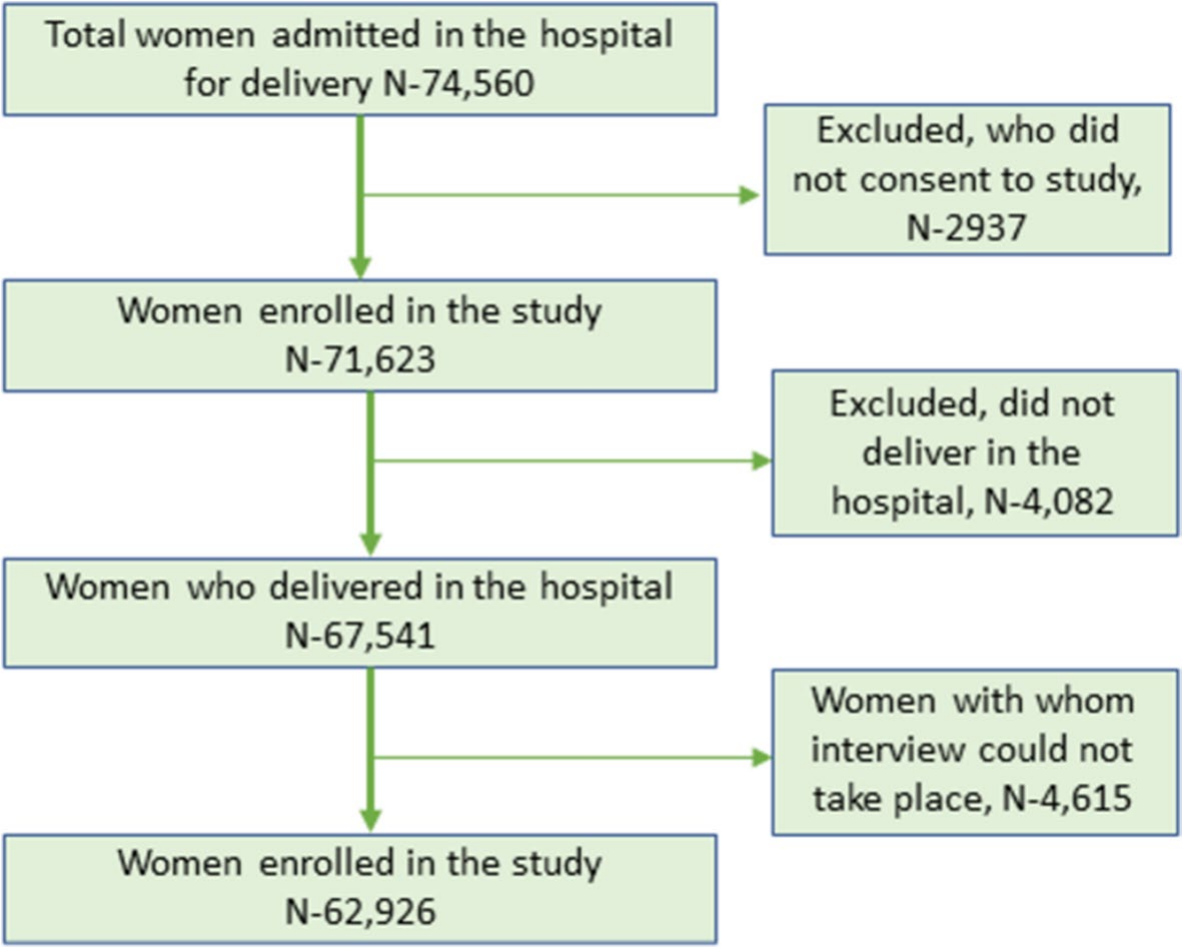
Participant flow

Of the 74,560 women admitted for delivery in the 11 hospitals during the study period, 62,926 of them consented to interview and were enrolled in the study (Fig. 2). Women with two or more previous births were more likely to participate in the interview than those who one or no previous birth; and no difference was seen in maternal age, gestational age and birth weight (supplementary Table 1). The mean age of the women enrolled was 23.92 ± 4.23 years, 37.8% of the women were from Brahmin ethnic group, 4.6% of the women were illiterate, 33.6% of them had one previous birth and 13.6% of them were preterm (supplementary Table 2).

### Magnitude of the mistreatment

#### Prevalence of mistreatment to newborns

Of the total 62, 926 women enrolled in the study, 99.2% did not experience physical, verbal, or sexual abuse during childbirth; 84.3% (*n* = 53,047) had no opportunity to discuss their concerns; 80.4% (*n* = 50,593) were not adequately informed on the care provided during childbirth; 42.1% (*n* = 26,492) were not counseled on exclusive breastfeeding; and 1.5% (*n* = 944) of them were refused care due to inability to pay during the postpartum period. Of the newborns enrolled in the study, 73.7% (*n* = 46,376) of their mothers were not counseled on neonatal danger signs, 59.9% (*n* = 37,693) were not examined at the time of discharge during the postpartum period, and 40.6% (*n* = 25,548) were not examined in the presence of women (Table 2).

**Fig. 3:**
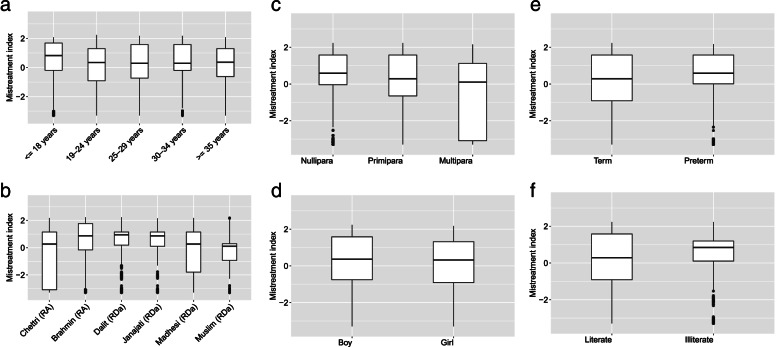
**a**-**f**. Univariate association of sociodemographic factors with mistreatment of women

Based on the first PCA, a continuous mistreatment index between − 3 to + 3 was generated. The box plot of the mistreatment index shows variation in the background characteristics of women and newborns. The prevalence of mistreatment also varied by the women’s age, ethnicity, literacy, and parity as well as the baby’s gestational age and sex. Maximum variation in the mistreatment index was observed among women from advantaged ethnic groups and infants born to women who had two or more previous births (Fig. 3).

In multivariate regression analysis, the covariates age, ethnicity, and preterm birth were significantly associated with the maternal mistreatment index even at a 5% level of significance. Women aged 35 years or older (β, − 0.3587; *p*-value, 0.000), 30–34 years (β, − 0.38013; *p*-value, 0.000), 25–29 years (β, − 0.28411; *p*-value, 0.000), and 19–24 years (β, − 0.18856; *p*-value, 0.000) were less likely to be mistreated than women aged 18 years or younger. Women from the advantageous (Brahmin) ethnic group were less likely to be mistreated (β, − 0.37987; *p*-value, 0.000) than those from the relatively advantageous (Chettri) ethnic group while, the women belonging to relatively disadvantageous ethnic groups of Muslim and Janjati were more likely to be mistreated (β, 0.18799; *p*-value, 0.000 and β, 0.27264; *p*-value, 0.000) when compared to the relatively advantageous (Chettri) ethnic group. Newborns who were born preterm (β, − 0.05988; *p*-value, 0.000) were less likely to be mistreated than those born at term (Table 3). There was hospital level heterogeneity for mistreatment during childbirth (supplementary Table 3).

**Table 3:** Simple and multiple linear regression modeling to assess the association between sociodemographic and obstetric characteristics with mistreatment of women

	Bivariate analysis		Multivariate analysis	
	β estimate	*p*-value	β estimate	*p*-value
Global intercept	–	–	0.15454	< 0.001
Ethnicity				
Intercept	−0.11823	0	–	–
Chettri, relatively advantageous	Reference			
Brahmin, relatively advantageous	−0.37463	0	−0.37987	< 0.001
Dalit, relatively disadvantageous	0.33653	0	0.29596	0
Janajati, relatively disadvantageous	0.27919	0	0.27264	0
Madhesi, relatively disadvantageous	−0.03575	0.19594	−0.06989	0.01217
Muslim, relatively disadvantageous	0.23603	0	0.18799	< 0.001
Maternal literacy				
(Intercept)	0.03563	0.06114		
Literate	Reference		Reference	
Illiterate	0.05475	0.04191	0.01303	0.63345
Maternal age				
Intercept	0.19597	0.000	–	–
18 years or younger	Reference		Reference	
19–24 years	−0.1874	0.000	−0.18856	0.000
25–29 years	−0.2374	0.000	−0.28411	0.000
30–34 years	−0.28214	0.000	−0.38013	0.000
35 years or older	−0.20265	0.00025	−0.35872	0.000
Parity				
Intercept	0.02936	0.00045	–	–
0 previous births	Reference		Reference	
1 previous birth	0.11673	0		
2 or more previous births	0.04273	0.00178	−0.03054	0.0398
Sex of baby				
Intercept	0.01348	0.183		
Boy	Reference		Reference	
Girl	−0.02932	0.04957	0.00313	0.78487
Preterm birth				
Intercept	−0.03326	0.00227		
No (≥37 weeks)	Reference		Reference	
Yes (< 37 weeks)	−0.06438	< 0.001	−0.05988	< 0.001

## Discussion

This study reports on how the prevalence of mistreatment among women during childbirth and postnatal period varied on the basis of women’s ethnicity, age, and gestational age of newborn. Physical, verbal, or sexual abuses during childbirth are very rare in public hospitals in Nepal. However, only one-fifth of women were adequately informed about the care or medical interventions provided during childbirth and had opportunity to discuss their concerns. Less than half of them were counseled on exclusive breastfeeding and only one-third of babies were examined in the presence of women. The prevalence and variance of mistreatment by sociodemographic characteristics (ethnicity, maternal age and gestation of newborn) suggests changes at an individual health worker as well as systemic level. This also calls for changes around social norms and expectations of women.

Despite the medical ethical guidance for all health workers to obtain consent from parents or caregiver to treat any infant, there is a poor adherence to this guidance [25]. A multi-centric health facility study on disrespect and abuse during childbirth in Addis Ababa showed the right to information, informed consent, and choice/preference were not protected in most of the women [26]. Almost half of the women were not asked for their consent or permission prior to any procedure for themselves or their newborns [26].

This study found that the risk of mistreatment of women increased among women from relatively disadvantaged ethnic group. In Nepal, caste and ethnicity remain the centerpiece of the social hierarchy [27]. Families from higher castes and relatively advantaged ethnic groups are more likely to get better quality of care [28], while discrimination is relatively more prevalent in daily life among disadvantaged ethnic groups [29]. Regular or frequent discrimination among disadvantaged ethnic group affects the self-reporting of mistreatment as they might have less expectations and normalize the undignified maternity care. A study in rural Northern India showed that women from scheduled tribes or other lower castes received more mistreatment during childbirth than women from non-scheduled or other castes [30]. A study in the United States on 2700 women showed that one in six women experienced mistreatment during childbirth [31]. The women’s race was associated with a higher likelihood of mistreatment.

Our study showed that women younger than 18 years of age are at higher risk of mistreatment during childbirth. A multi-country study done in four countries showed that almost one-third of the surveyed women experienced physical or verbal abuse, stigma, or discrimination [32]. Younger age (15–19 years) was the primary determinant of mistreatment [32]. Adolescent pregnancy is stigmatized in Nepal [32] which might have contributed to health workers’ attitude towards young to be mother as well as health workers find them. The higher mistreatment among young pregnant women might also be because these women are less educated and are less aware of their rights during childbirth [33] which makes them easy target of frustrated health care providers. A systematic review of 14 studies on disrespect and abuse of women during childbirth in Nigeria showed different types of abuse and risk factors, which were influenced by the women’s age and lack of education [33].

### Methodological considerations

Team of skilled researchers is the strength of this study having an experience of consistently conducting the measurement study in multiple hospitals in Nepal with large sample size. Another strength is the independent research nurses hired for data collection were not prior exposed to or have worked in those hospitals which has reduced subjective bias to some extent.

However, there are several limitations. First, this is a nested study which was carried out within the large observational study. And the aim of the main study was to improve the Quality of Care (QoC) and perinatal outcomes of babies. Hence, the interventions to improve QoC (e.g. training/mentoring the providers) may have helped to reduce mistreatment particularly the standards of care, as compared to non-intervened hospital where QoC was not implemented. Thus, the results may underestimate of the actual situation in the hospitals across the country. Second, we conducted interviews inside the hospital which might have influenced the response of the postnatal women leading to underreporting true childbirth experience. Third, large number of values are missing for women’s literacy and sex of newborns, and the missing values are not randomly distributed to be excluded from the analysis, so imputation was done. Fourth, not all women consented to provide exit interview, 15.6% declined to interview at the time of discharge. However, there was no difference in basic demographic characteristics. Fifth, some level of interviewer bias might have existed due to the ethnicity, attitude and body language of the interviewer. Finally, women rarely reported abuse and might be under reported as they provide interview in the hospital. Qualitative research in some secure setting might provide accurate information on abuse.

Despite these limitations, this study provides one of the first and largest rigorous documentation of mistreatment of women and newborns immediately after birth in health facilities, and it can provide the evidence for further research, hospital level intervention as well as policy advocacy for respectful maternity care (RMC). Study done in similar low-income settings in Africa and Asia have shown mistreatment of women during childbirth and factors associated with mistreatment [30–32]. Our study provides new evidence on mistreatment of women based on the social class they belong to.

## Conclusion

Mistreatment of women and newborns during childbirth in health facilities was found across different components, but only very few women reported physical, verbal, or sexual abuse. The prevalence of mistreatment varied gestational age of the baby. Young women and those from disadvantaged ethnic groups are at increased risk of mistreatment. There was high rate of mistreatment reported in terms of receiving inadequate information before the care and no opportunity for discussion during childbirth. New mothers reported of not being counselled during the postnatal period referring to major communication gap between health care provider and women. Strengthening health system and improving the readiness of health workers as well as the women in the community are crucial to establish trust with the health care system. This will improve attitude and provision of care resulting in better childbirth experience.

## Supplementary Information

**Additional file 1: Supplementary Table 1.** Demographic and obstetric characteristics of women who consented and those who did not consent.

**Additional file 2: Supplementary Table 2.** Demographic characteristics of women who participated in the study.

**Additional file 3: Supplementary Table 3.** Hospital level heterogeneity of mistreatment during childbirth and postnatal period.

## Data Availability

The datasets used and/or analysed during the current study are available from the corresponding author on reasonable request.

## Abbreviations

HCP: Health care professionals
WHO: World Health Organization
ENAP: Every Newborn Action Plan
STROBE: Strengthening the Reporting of Observational Studies in Epidemiology
CEONC: Comprehensive and Emergency Obstetrics and Neonatal Care
SNCU: Special Newborn Care Unit
CS-PRO: Census and Survey Processing System
PCA: Principal component analysis
RMC: Respectful Maternity Care
