# Supplementary material for: Mistreatment during childbirth and postnatal period reported by women in Nepal —a multicentric prevalence study
Source: BMC Pregnancy Childbirth. 2022 Apr 14;22:319. doi: 10.1186/s12884-022-04639-6 (PMC9011987; doi:10.1186/s12884-022-04639-6)
Supplement: Supplementary file 1 — Additional file 1: Supplementary Table 1. Demographic and obstetric characteristics of women who consented and those who did not consent. [file 12884_2022_4639_MOESM1_ESM.docx]

|  | Women consented to interview (n=62,962) | Women who did not consent to interview (n=9634) | p-value |
| --- | --- | --- | --- |
| Age in years, mean±SD | 23.9±4.2 | 24.0±4.4 | 0.412* |
| Infant's birth weight in gram, mean±SD | 2881.7±626.1 | 2628.9±881.1 | 0.127* |
| Gestational age in weeks, mean±SD | 38.2±3.1 | 36.8±4.7 | 0.142* |
| Women's parity |  |  |  |
| No previous birth | 31745 (51.1%) | 4567 (47.4%) | 0.021** |
| One previous birth | 20846 (33.6%) | 3054 (31.7%) | 0.081** |
| Two or more previous birth | 9512 (15.3%) | 2013 (20.9%) | 0.016** |
