# Supplementary material for: Mistreatment during childbirth and postnatal period reported by women in Nepal —a multicentric prevalence study
Source: BMC Pregnancy Childbirth. 2022 Apr 14;22:319. doi: 10.1186/s12884-022-04639-6 (PMC9011987; doi:10.1186/s12884-022-04639-6)
Supplement: Supplementary file 2 — Additional file 2: Supplementary Table 2. Demographic characteristics of women who participated in the study. [file 12884_2022_4639_MOESM2_ESM.docx]

|  | Percent (%) |
| --- | --- |
| Ethnicity |  |
| Dalit (n = 9,867) | 15.7 |
| Janajati (n = 18,421) | 29.3 |
| Madhesi (n = 6,034) | 9.6 |
| Muslim (n = 1,976) | 3.1 |
| Brahmin (n = 2,3806) | 37.8 |
| Chhetri (n = 2,822) | 4.5 |
| Women’s literacy |  |
| Literate (n = 60,022) | 95.4 |
| Illiterate (n = 2,904) | 4.6 |
|  |  |
| Age (mean ± SD) years | 23.92 ± 4.23 |
| Age category |  |
| 18 years or younger (n = 4,750) | 7.5 |
| 19–24 years (n = 33,264) | 52.9 |
| 25–29 years (n = 17,532) | 27.9 |
| 30–34 years (n = 5,816) | 9.2 |
| 35 years or older (n = 1,564) | 2.5 |
| Parity |  |
| No previous births (n = 31,828) | 50.6 |
| 1 previous birth (n = 21,125) | 33.6 |
| 2 or more previous births (n = 9,973) | 15.8 |
| Mode of birth |  |
| Spontaneous vaginal birth (n = 46,739) | 74.3 |
| Assisted vaginal birth (n = 2,505) | 4 |
| Caesarean birth (n = 12,501) | 19.9 |
| Not recorded (n = 358) | 0.6 |
| Missing (n = 823) | 1.3 |
| Sex |  |
| Boy (n = 34,116) | 54.2 |
| Girl (n = 28,810) | 45.8 |
| Preterm birth (<37 weeks) |  |
| Term birth (n = 54,360) | 86.4 |
| Preterm birth (n = 8,566) | 13.6 |
| Total | 100 |
| Low birth weight |  |
| Weight 2,500 g or heavier (n = 54,741) | 87 |
| Weight less than 2,500 g (n = 8,185) | 13 |
