# Supplementary material for: Mistreatment during childbirth and postnatal period reported by women in Nepal —a multicentric prevalence study
Source: BMC Pregnancy Childbirth. 2022 Apr 14;22:319. doi: 10.1186/s12884-022-04639-6 (PMC9011987; doi:10.1186/s12884-022-04639-6)
Supplement: Supplementary file 3 — Additional file 3: Supplementary Table 3. Hospital level heterogeneity of mistreatment during childbirth and postnatal period. [file 12884_2022_4639_MOESM3_ESM.docx]

| Hospital | X. Intercept. |
| --- | --- |
| Hospital 1 | -0,1423 |
| Hospital 2 | 1,0764 |
| Hospital 3 | -0,2813 |
| Hospital 4 | -1,316 |
| Hospital 5 | 1,1682 |
| Hospital 6 | -1,127 |
| Hospital 7 | -0,2132 |
| Hospital 8 | 1,2102 |
| Hospital 9 | -2,4926 |
| Hospital 10 | 1,4168 |
| Hospital 11 | 0,7007 |
